# An Audit of Preoperative Informed Consent in Surgical Patients at a Tertiary Care Hospital in Lahore, Pakistan

**DOI:** 10.7759/cureus.50122

**Published:** 2023-12-07

**Authors:** Muhammad Umer Shafique, Muhammad Salman Saleem, Maryam Saghir, Mohammad Saad Javaid, Mohammad Saad, Ahmad Sadiq, Hafiz U Shibli, Muhammad Ahmad Khalid, Farhan Saleem

**Affiliations:** 1 Medicine, Lahore General Hospital, Lahore, PAK; 2 Surgery, Services Hospital, Lahore, PAK; 3 General Medicine, Lahore General Hospital, Ameer-ud-Din Medical College, Lahore, PAK; 4 Internal Medicine, Lahore General Hospital, Lahore, PAK; 5 Internal Medicine, Lahore General Hospital, Ameer-ud-Din Medical College, Lahore, PAK; 6 Medicine, Ameer-ud-Din Medical College, Lahore, PAK; 7 Orthopaedic Surgery, Lahore General Hospital, Lahore, PAK

**Keywords:** decision-making paradigm, healthcare education, anesthesia risks, surgical patient survey, tertiary care hospital, surgical procedures, medical ethics, patient rights, clinical practice, informed consent

## Abstract

Informed consent plays a crucial role in modern clinical practice, representing a fundamental aspect of patient rights and medical ethics. The purpose of informed consent is to ensure that patients fully comprehend the procedures to which they are providing consent and the recognition that the surgeon is not guilty of battery. Moreover, clinicians safeguard themselves against potential repercussions by documenting the risks adequately conveyed to patients before performing surgery. Therefore, the significance of informed consent cannot be overstated. This survey encompassed patients from various surgical departments who underwent surgery in April 2023 at a tertiary care hospital. For the survey participants above the age of 18 were selected undergoing either emergency or elective surgical procedures. The survey employed a structured questionnaire for interviews, assessing whether patients had given informed consent before surgery. The questionnaire also inquired whether patients received information about the diagnosis, proposed surgical procedure, associated risks, and any available alternative treatment options. Furthermore, patients were asked about the proposed anesthesia type and whether the associated risks were communicated to them before the surgery.

A random selection of 50 patients was done for this study, and the process of block randomization was used with the help of a computer app to reduce bias and allow the representation of the various surgical subspecialties present in the tertiary care hospital. No evidence of consent being taken was present in two patients(4%) or the document on which the consent was signed was not present in the file. Only 48% of the patients acknowledged that they fully understood the provided information. While 60% of the patients were informed about the type of anesthesia proposed, a mere 8% were provided information regarding anesthesia risks. None of the patients in the emergency setting signed the consent form themselves, regardless of their capability to do so. Conversely, only 24% of the patients in the elective setting signed the consent form themselves. The study revealed that the quality of informed consent signing in this tertiary care hospital is below average. Healthcare professionals, including doctors and staff, need education regarding the importance of informed consent and the patient's right to comprehend any procedure or intervention to which they are subjected. A shift in the paradigm of decision-making about a patient's health needs to emphasize that the patient is the most critical entity in these decisions.

The main aim of the study is twofold, primarily we want to analyze the existing method of taking informed consent by comparison with the guidelines and check whether the current practice of informed consent achieves its goal of involving the patients in their treatment. Secondarily, we want to discuss the effect that patient-doctor communication might have on the delivery of the above-mentioned information.

## Introduction

Informed consent is a procedure in which a patient with adequate mental and physical ability receives necessary information about their condition so that he or she can participate in decisions regarding their medical care. It is generally agreed that a discussion of the procedure’s nature, plausible alternatives to the suggested treatment, and the pertinent risks and benefits of the proposed procedure are all part of an informed consent [1]. The patient must comprehend the facts supplied, and the consent must be given voluntarily [2]. Both the information presented and the patient’s understanding are crucial. Therefore, the information offered should be explained in simple terms and understandable to the patient.

In the West, there has been a paradigm change, with more patients desiring to be thoroughly informed about procedural possibilities and dangers [3]. Unfortunately, clinical practice in our setup does not emphasize enough the relay of medical information to the patients and their relatives. This study aims to evaluate the ongoing process of obtaining informed consent from patients who are undergoing elective or emergency procedures in a tertiary care hospital in the public sector. Additionally, the understanding of information and facts about the procedure from the point of view of the individual giving consent is taken into account. Both these factors are dependent on a number of factors such as patient-doctor communication, among others, which will be discussed.

## Materials and methods

The survey was carried out at a tertiary care university hospital in April 2023. The patients included in the study were over the age of 18 having undergone emergency or surgical procedures in departments of urology, general surgery, obstetrics and gynecology, and neurosurgery. Permission to conduct the survey was obtained from the heads of departments of each unit. The interviews were conducted in the immediate postoperative period as soon as the patients were able to comfortably answer the interviewer’s questions and any individual who had signed the consent was also present. Patients experiencing pain, having nasogastric tubes, or encountering any other postoperative complications were excluded from the survey. A structured questionnaire was used for the survey. Approval of the study was taken from the Ethics Committee of Lahore General Hospital, Lahore.

## Results

The study involved the random selection of 50 postoperative patients through block randomization with a computer app. Patient demographics are presented in Table 1. In two cases (4%), patient consent was not obtained. The two cases without consent were both in the Department of General Surgery. In none of the cases was consent obtained by the surgeon actually performing the surgical intervention.

**Table 1:** Demographic data ASA = American Society of Anesthesiologists N = Total Number of Patients (50) (p value considered significant when p<0.05)

CHARACTERISTICS	NUMBER OF PATIENTS (N=50)	PERCENTAGE (%)
GENDER		
Male	31	62%
Female	19	38%
ASA STATUS		
ASA 1	36	72%
ASA 2	11	22%
ASA 3	3	6%
TYPE OF SURGERY		
General Surgery	22	44%
Urology	8	16%
Orthopedics	5	10%
Neurosurgery	5	10%
Gynecology/Obstetrics	10	20%
NATURE OF SURGERY		
Elective	34	68%
Emergency	16	32%

Consent was taken in 41 of the cases (82%) in the patient’s mother language; however, only 16 patients (32%) were able to fully comprehend the information that was given to them. Consent was mostly taken on a pre-printed form (90%, 45 patients), while the rest were handwritten (10%, five patients). None of the patients in the emergency setting signed the consent form themselves, while in the elective setting, 12 patients (24%) signed the consent form themselves. In other cases, the consent forms were signed by family members (52%, 26 patients), spouses (20%, 10 patients), and offspring (4%, two patients). Table 2 gives information as to the number and percentage of patients who were informed about the alternate treatment options, complications of surgical procedures, and types and complications of anesthesia.

**Table 2:** Characteristics of informed consent N = Total Number of Patients (50) (p value considered significant when p<0.05)

CHARACTERISTICS	NUMBER OF PATIENTS (N=50)			
	Patients informed	Percentage of patients informed	Patients not informed	Percentage of patients not informed
Alternate treatment options	22	44%	28	56%
Complications of surgical procedure	12	24%	38	76%
Type of anesthesia	30	60%	20	40%
Complications of anesthesia	4	8%	46	92%

## Discussion

From the perspective of law, as well as that of the code of ethics, physicians are required to take informed consent from their patients. This consent needs to be taken before beginning any form of treatment, and surgical procedures/interventions are also included in this gambit [4]. This practice ensures the active participation of the patient and their family in the treatment process while respecting the patient's right to understand their treatment plan [5,6]. In the West, there has been a growing trend of involving patients more in decisions regarding their treatment, largely due to an increase in patients' knowledge of their rights, facilitated by media coverage and improved education.

**Figure 1:**
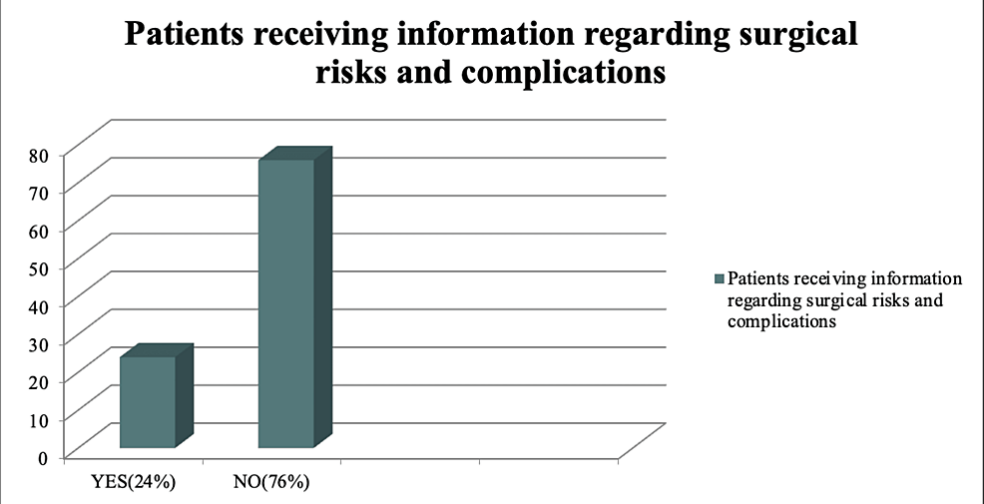
Information regarding surgical risks and complications Total Number of Patients = 50 Yes (24%) = 12 patients received information regarding surgical risks and complications No (76%) = 38 patients did not receive information regarding surgical risks and complications

**Figure 2:**
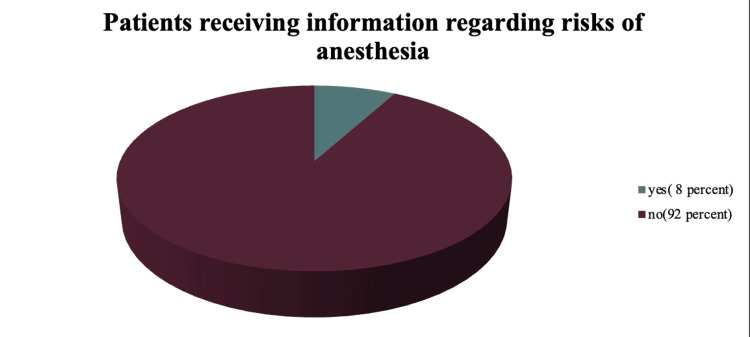
Information regarding the risks of anesthesia Total Number of Patients = 50 Yes (8%)= 4 patients received information regarding the risks of anesthesia No (92%) = 46 patients did not receive information regarding the risks of anesthesia

In this survey, almost all patients had the signed consents in their files, signed by either them or their relatives; however, only 32 individuals who signed the document (64%) claimed to have fully understood all the information provided to them. Upon further questioning, the remaining individuals cited various reasons for not comprehending the information, primarily a communication gap between the doctor and the patient, particularly for those with limited education. While 42 individuals received information regarding the disease diagnosis (84%), the majority did not receive information about the surgical procedure itself or the associated risks and complications, as depicted in Figure 1. Only four patients (8%) received any information regarding the risks and complications of the anesthesia planned for the surgery (Figure 2). A similar study was conducted by Amin et al., in which they found that 71.5% of the participating patients in their study were informed about the condition they were suffering from and 45% of the patients received information about the nature of the surgery/intervention that was proposed to treat the condition [7].

A total of 38 patients (76%) in our audit were not provided with adequate information regarding the risks and associated complications of the surgical procedure they underwent. In a study conducted by Vessey et al., it was found that the majority of patients understood the reason for the surgery being conducted; however, 28 out of the 49 patients (57.1%) who planned for the surgery due to acute abdomen were not aware of the complications that the surgery entailed before the procedure began [8]. On the other hand, another study showed that 69.3% of patients did not receive any information about the risks of surgery before the procedure [9]. The most commonly cited reason for not providing complete information about the diagnosis and treatment complications is the fear that it will increase the patient's anxiety. This view is ethically wrong, as the doctor makes assumptions about the patient's behavior and, in doing so, usurps their right to knowledge of their condition and proposed treatment. Communication skills on the part of the doctor are key factors in obtaining ideal informed consent from the patient. These skills help reduce the patient's anxiety, and they are better able to retain information provided to them by health practitioners, and then they can make better well-informed decisions regarding their health. Good verbal communication is helpful in maintaining a level of trust and understanding between the patient and the doctor, as seen in a study by Ranjan et al. and Bone et al. [10,11]. Similarly, showing empathy and sympathy while explaining the details of the patient's conditions and the interventions proposed, along with the use of nonverbal cues, such as simple gestures, improve the level of satisfaction reported by the patients [12,13].

A study done by Larobina et al. completely nullifies this totally baseless impression. This study reports that the majority of the patients wanted to know all the risks associated with the surgery that was going to be performed on them. These surgeries included coronary artery bypass graft (CABG) surgery or percutaneous coronary intervention (PCI), and none of the patients undergoing these procedures identified the risks explained to them as a reason to rethink the surgery [14]. Observation has shown that, while most patients are informed about their diagnosis and the planned treatment, the complications and risks of the said treatment are often not divulged [15].

A total of 30 patients (60%) in our study did receive some, if not complete, information regarding the type of anesthesia they were administered. However, next to none (8%, four patients) were given information regarding the different risks and complications that normally accompany the administration of anesthesia. In another study, a similar thing was noted, that is, a very low proportion of people being informed regarding the complications associated with anesthesia [15]. Despite the presence of a separate printed form for anesthesia consent, it is usually assumed that a patient consenting to surgery has also consented to the anesthesia, and little to no effort is made by the anesthetists to inform the patient regarding the anesthesia being administered to them and the risks and complications associated with it. It is regarded as unacceptable for doctors other than anesthetists to divulge information regarding anesthesia, which they seldom do in our medical setting. Hence, there is a dire need for the re-education of anesthetists regarding the process of informed consent and to revise the guidelines regarding informed consent, mainly pertaining to anesthetists.

Most of the consents were taken on a printed form, with two (4%) being handwritten. Documentation plays an integral role in all medical practice, especially surgical procedures. It safeguards both the patients' and the doctors' rights and, in any legal complication, protects the doctor from wrongful accusations. We asked patients about the importance of documentation, and they overwhelmingly favored it. In a similar study in Karachi, 61% of the population agreed on the importance of documentation [16]. It is important to note that, while in the West, it is important that the patients consent to the medical procedure themselves unless they are unable to. In our tertiary care setting, only 12 patients (24%) signed on the consents themselves; others who signed included family members (52%, 26 patients), spouses (20%, 10 patients), and offsprings (4%, two patients).

Limitations

During the study, attempts have been made to eliminate any bias that may be present by ensuring anonymity and a layered organizational structure for the collection of data. However, still there may be some selection bias present in the study as only certain surgical departments consented for the study to take place in their departments. Furthermore, the study does not include patients that, unfortunately, may have expired during or after the procedures within the time frame in which the study was conducted. The sample size is limited due to the availability of limited patients due to COVID restrictions, which, in an ideal case, could have increased.

## Conclusions

The current practice of obtaining informed consent falls below the expected standards, and patients' rights to knowledge about their diagnosis and risks of complications are not being fully fulfilled. There is a dire need for improvement in the status quo, necessitating surgical and anesthesia departments to establish strict guidelines for doctors to adhere to. Promoting the attitude of obtaining comprehensive informed consent from the patient will have a dual-pronged effect, benefiting both the doctors and the patients. The patient's right to knowledge will be safeguarded; on the other hand, the doctors will feel more secure legally if they document everything effectively and efficiently. Moreover, this will contribute to forming a better bond between the doctor and the patient.
